# Changing Expression Profiles and Inclination to Competing Endogenous RNA Networks on MAPK Signaling Pathways of Human Adipose-Derived Stem Cells in a Direct Current Electric Field

**DOI:** 10.1155/2020/7134719

**Published:** 2020-11-06

**Authors:** Mingzhu Jin, Yujing Zhang, Yuanyuan Bian, Ruiqun Qi, Xinghua Gao

**Affiliations:** Department of Dermatology, The First Hospital of China Medical University and Key Laboratory of Immunodermatology, Ministry of Health and Ministry of Education, Shenyang 110001, China

## Abstract

Adipose-derived stem cells (ADSCs) are an abundant cell source and provide an easy way to harvest mesenchymal stem cells, which are the focus of considerable attention in regenerative medicine. Electric fields (EF) play roles in many biological events and have been reported to promote cell proliferation, migration, and differentiation. In this study, ADSCs were treated with a direct current electric field (DCEF) of either 0 (control group) or 300 mV/mm (EF group) for six hours. RNA screening and analysis revealed that 66, 164, 26, and 1310 circRNAs, lncRNAs, miRNAs, and mRNAs, respectively, were differentially expressed in the DCEF-treated ADSCs compared with untreated ADSCs. Differentially expressed mRNAs were enriched in the MAPK signaling pathway, TNF signaling pathway, and some other pathways. ANXA1, ERRFI1, JAG1, EPHA2, PRR9, and H2AFY2 were related to the keratinocyte differentiation process. Competing endogenous RNA (ceRNA) networks were constructed on the basis of genes in the MAPK signaling pathway. Twenty-one RNAs in the above networks were randomly chosen, and their expression was validated using qRT-PCR, which showed the same expression trends as the RNA sequencing analysis. The MAPK signaling pathway is of great importance in the ADSC processes that occur in a DCEF, including keratinocyte differentiation. Several ceRNAs may participate in the regulation of MAPK signaling. This study may give new insight into the proliferation, migration, and differentiation of ADSCs, which will be valuable for tissue engineering and regenerative medicine.

## 1. Introduction

Mesenchymal stem cells (MSCs) are a population of adult stem cells with self-renewal properties and multilineage differentiation capacity. Compared with embryonic stem cells (ESCs) and induced pluripotent stem cells (iPSCs), which have the limitations of ethical issues and tumorigenicity, MSCs have gained extensive attention in regenerative medicine [1, 2]. MSCs have been reported to be isolated from a number of sources, including the bone marrow, adipose tissue, umbilical cord, and placental tissue [3]. Adipose-derived stem cells (ADSCs) are advantageous due to their abundant cell sources and ease of harvesting [4].

Electric fields (EFs) are necessary to maintain homeostasis in cells and participate in many biological events ranging from embryogenesis to tissue healing [5]. EF has several potential advantages over other stimuli as no immunogenic bioagent or complicated equipment is involved [6, 7]. At present, various methods of electrical stimulation have been applied in vitro, including direct current electric field (DCEF). DCEF can not only affect the survival and proliferation of many cell types but also change cell migration and morphology. Previous studies have shown that DCEF can trigger morphological changes in ADSCs and force cells to align vertically to the EF vector or even induce them to migrate to the cathode, which is also referred to as galvanotaxis [8].

Multiple noncoding RNA species, including small noncoding RNAs such as miRNAs, pseudogenes, long noncoding RNAs (lncRNAs), and circRNAs, may possess competing endogenous RNA (ceRNA) activity. In particular, lncRNAs and circRNAs can bind some active miRNAs and indirectly regulate the expression of other transcripts targeted by the same set of miRNAs [9–11]. To our knowledge, there have been no studies on transcriptome sequencing and ceRNA network prediction of ADSCs in an EF.

In this study, we used whole-transcriptome sequencing technology to investigate the RNA expression profile in ADSCs stimulated by DCEF of 0 and 300 mV/mm for 6 h, respectively. Differentially expressed mRNAs, miRNAs, lncRNAs, and circRNAs were identified. The differentially expressed mRNAs were primarily enriched in the MAPK and TNF signaling pathways. ANXA1 and some other mRNAs were related to keratinocyte differentiation process. Moreover, we predicted the ceRNA networks based on the selected genes in the MAPK signaling pathway. Finally, 21 RNAs were randomly chosen, and their expression profiles were validated using qRT-PCR.

## 2. Materials and Methods

### 2.1. Ethics Statement

The study was approved by the Ethics Committee of the First Hospital of China Medical University and complied with the Helsinki Declaration.

### 2.2. ADSC Isolation and Culture

Fat tissue was obtained from three donors who underwent plastic surgery, after they provided signed informed consents. Lipoaspirates were washed 3 times with phosphate-buffered saline (PBS; Biological Industries, CT, USA) to remove erythrocytes and enzymatically digested with 0.1% collagenase I (Solarbio, Beijing, China) for 1 h at 37°C. After neutralizing the cells with 10% fetal bovine serum (FBS; Biological Industries), centrifugation, and PBS washing, cells were resuspended and cultured in DMEM/F-12 medium (Biological Industries) supplemented with 10% FBS and 1% penicillin/streptomycin (Biological Industries) at 37°C in a 5% CO_2_ incubator. The medium was changed every 3 days until 90% confluence was achieved. ADSCs were detached with 0.25% trypsin-EDTA (Biological Industries) then passaged. ADSCs from passages 3–5 were used in this study.

### 2.3. Authentication of ADSCs

Passage 3 ADSCs were digested by trypsin-EDTA and washed with PBS, then incubated with fluorescein isothiocyanate- (FITC-) conjugated and phycoerythrin- (PE-) conjugated antibodies, including anti-CD34-FITC, anti-CD44-FITC, anti-CD90-FITC, anti-CD45-PE, anti-CD73-PE, anti-CD105-PE, PE-labeled mouse IgG1 Kappa, and FITC-labeled mouse IgG1 Kappa (BD Pharmingen, USA) at 4°C for 40 min. We used flow cytometry (LSRFortessa, BD Biosciences, USA) to record and analyze the data. At least 1 × 10^4^ cells were analyzed per test.

Briefly, 12-well plates were pretreated with gelatin (Cyagen, USA) to enhance adherence. Then, to confirm the multilineage differentiation ability of ADSCs, passage 3 cells were seeded at a density of 1 × 10^4^ cells/well and cultured until 60–70% confluence was achieved. The medium of some of the wells was then changed to mesenchymal stem cell osteogenic differentiation medium (Cyagen, USA) according to the manufacturer's instructions. The medium was changed within 72 h. For adipogenic differentiation, we used mesenchymal stem cell adipogenic differentiation medium (Cyagen, USA) according to the manufacturer's instructions. Two to four weeks later, the cells were stained with alizarin red S (Solarbio, China) and oil red O (Cyagen, USA) separately to identify osteogenic and adipogenic differentiation, respectively.

### 2.4. Electric Field Application

ADSCs after five passages were exposed to a DCEF of 300 mV/mm in our laboratory-made equipment described in detail previously [12, 13]. In brief, cells were seeded at 3 × 10^4^/cm^2^ on a 100 mm petri dish, which was treated before seeding with poly-D-lysine (Beyotime, China) for 5 min and then left to dry for at least 30 min. Then, the cells were put back into the incubator for at least 6 h to achieve attachment. Coverslips were attached using high-vacuum silicone grease (Dow Corning, USA) to form a small chamber with silicone grease separating the petri dish into two reservoirs. Fresh medium was added into the petri dish, and the two separated reservoirs were allowed to connect. The petri dish was returned to the incubator for at least 12 h to allow for cell recovery. The medium was changed, and HEPES buffer (Solarbio, China) was added to reach 25 mM to maintain pH stability. Then, 2% agarose (Sigma, USA) salt bridges were placed on both sides of the petri dish, while the other side of salt bridges put in Steinberg's solution, connected with silver wires (Alfa Aesar, USA) to a direct current power supply (Maisheng, China). The voltage of the chamber was measured and adjusted every hour to reach the set chamber EF strength.

### 2.5. RNA Extraction and Quality Control

Total RNA was extracted using TRIzol reagent (Thermo Scientific, MA, USA). RNA integrity and gDNA contamination were measured using electrophoresis with denatured agarose (Sangon Biotech, Shanghai, China). The purified RNA concentration was detected by NanoDrop ND-1000 (Thermo, USA).

### 2.6. Library Preparation and RNA Sequencing

RNA sequencing was accomplished by Cloud-Seq Biotech (Shanghai, China). See the Supplementary Methods for additional information (available here).

### 2.7. Identification of RNAs and Differential Expression Analysis

RNA identification was accomplished by different methods according to RNA species. Differential expression of RNA was determined by fold change and *p* value. Please see the Supplementary Methods for additional information (available here).

### 2.8. GO and KEGG Pathway Analysis

We employed the GO and KEGG pathway analysis tool to identify the differentially expressed mRNAs. A *p* value of <0.05 was used as the threshold of significant enrichment.

### 2.9. ceRNA Network Construction

StarBase (v2.0) [14] and Cytoscape (v3.7.1) were applied to identify and construct the predicted ceRNA networks.

### 2.10. Validation of RNA Expression by qRT-PCR

For circRNAs, lncRNAs, and mRNAs, the annealing mixture was incubated at 65°C for 5 min and put on ice for 2 min, then mixed to compose a reverse transcription mixture, followed by incubation at 50°C for 60 min and 70°C for 15 min. For miRNAs, the reverse transcription mixture was incubated at 16°C for 30 min, 42°C for 40 min, and 85°C for 5 min. Using SYBR Green master mix (CloudSeq, China), PCR was carried out at conditions of 95°C for 10 min, followed by 40 cycles of 95°C for 10 s and 60°C for 1 min. U6 was chosen as the miRNA reference while GAPDH was used as the reference gene for the other three RNA groups. Supplementary Table S1 displays the primers for RNAs and control genes.

### 2.11. Statistical Analysis

GraphPad Prism was used for statistical analysis. The paired Student's *t*-test was used to compare the RNA expression profiles of the experiment and control groups. A *p* value of <0.05 was considered statistically significant.

## 3. Results

### 3.1. ADSC Authentication

**Figure 1:**
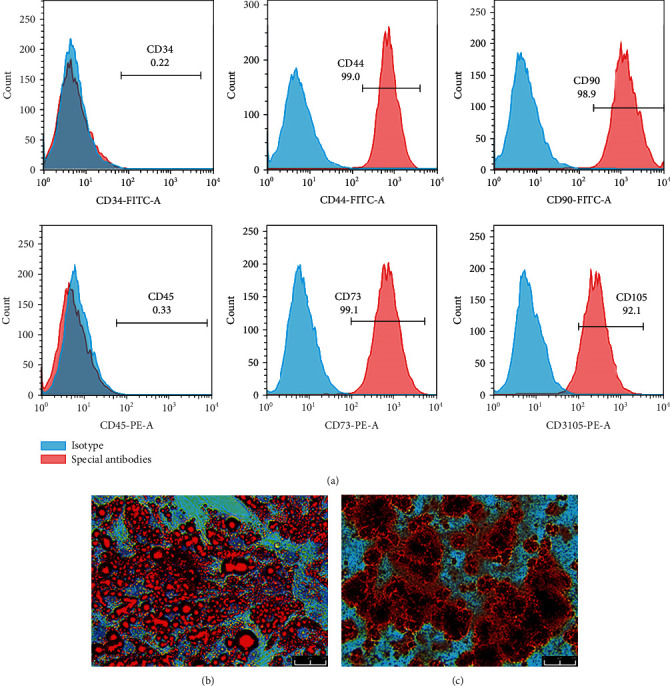
Characterization of adipose-derived stem cells (ADSCs): (a) expression of the characteristic surface markers of ADSCs shown by flow cytometry; (b) oil red O staining showing adipogenic induction after 14 days of induction; (c) alizarin red S staining showing osteogenic induction after 21 days of induction.

The collagenase-digesting method was used to isolate ADSCs. Expanded cells were authenticated by flow cytometry and multilineage differentiation. Characteristic ADSC surface markers, including CD34, CD44, CD45, CD73, CD90, and CD105, were chosen. The hematopoietic lineage marker CD34 and the leukocyte marker CD45 were not expressed while the other 4 markers were expressed, confirming that the cells we obtained and used were ADSCs (Figure 1(a)). To confirm the multilineage potential, adipogenic differentiation and osteogenic differentiation were carried out in differentiating media. Figures 1(b) and 1(c) show the adipocytes and osteoblasts that differentiated from the ADSCs.

### 3.2. Differential Expression Analysis of circRNAs, lncRNAs, miRNAs, and mRNAs

**Figure 2:**
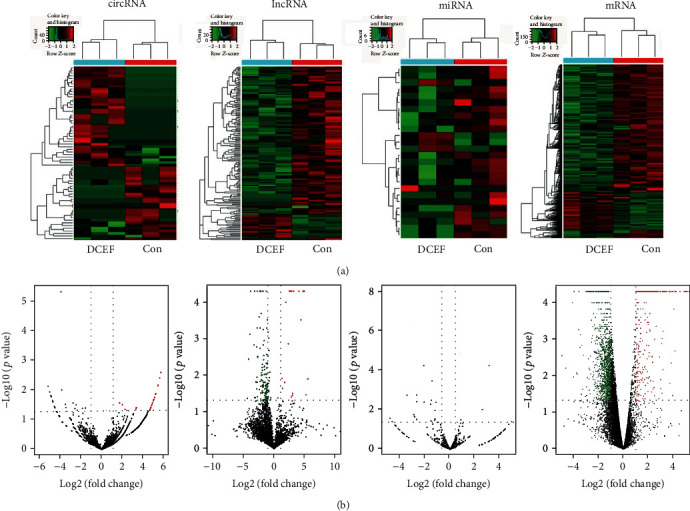
Expression profiles of circRNAs, lncRNAs, miRNAs, and mRNAs. Differential ncRNAs and mRNAs in the three paired EF and control cell groups are shown using (a) heat map and (b) volcano plot.

ncRNAs and mRNAs that were differentially expressed in cells of the DCEF group (*n* = 3) relative to those in the control group are indicated in a heat map and a volcano map (Figure 2).

Finally, 8944 circRNAs were detected, among which 2234 were novel ones. Of these, we identified 66 significantly dysregulated circRNA transcripts with 38 upregulated and 28 downregulated transcripts. Of the 18564 lncRNAs detected, 164 were significantly differentially expressed, including 24 upregulated lncRNAs and 140 downregulated lncRNAs. A total of 26 differentially expressed miRNAs were screened with 5 upregulated in the DCEF group and 21 downregulated, among 678 detected miRNAs with 117 novel ones. Among 17,252 detected mRNAs, 1310 differentially expressed mRNAs were screened, of which 274 were upregulated and 1036 were downregulated in the DCEF group.  Table 1 displays the circRNAs, lncRNAs, miRNAs, and mRNAs with the highest log_2_ fold change.

### 3.3. GO and KEGG Pathway Analysis in mRNA Differential Expression

**Figure 3:**
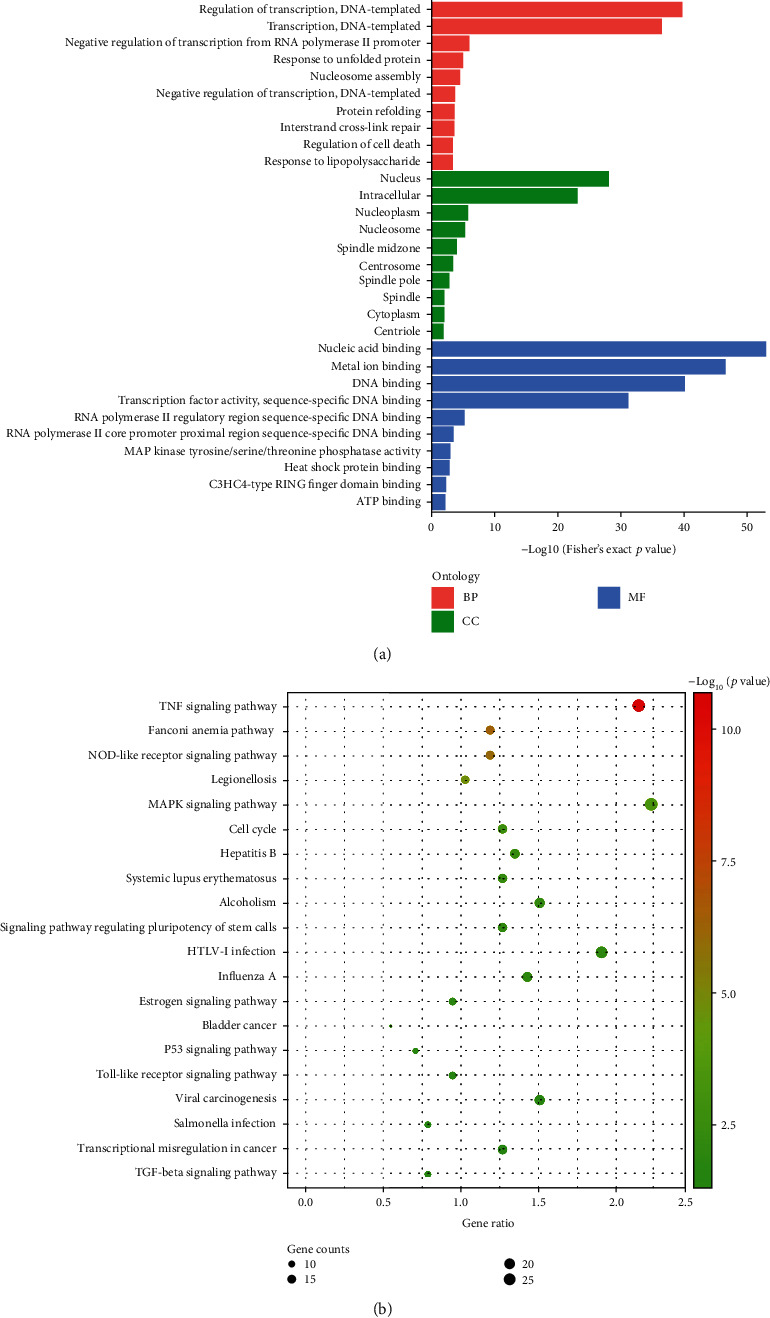
GO and KEGG pathways of differentially expressed mRNAs (a) GO term enrichment and (b) KEGG pathway analysis for the differentially expressed mRNAs.

To study the expression of upregulated and downregulated genes, we enriched these genes under the GO terms BP (biological processes), CC (cellular components), and MF (molecular functions) (Figure 3(a)). When the upregulated mRNAs were enriched, mRNAs involved in protein refolding and regulation of the p38MAPK cascade in BP; clathrin-sculpted vesicle, endocytic vesicle lumen in CC, and MAP kinase tyrosine/serine/threonine phosphatase activity; MAP kinase phosphatase activity in MF were the terms showing the highest level of enrichment. When the downregulated mRNAs were enriched, protein localization to kinetochore in BP, condensed chromosome outer kinetochore in CC, endodeoxyribonuclease activity, and producing 5′-phosphomonoesters in MF were the terms showing the highest level of enrichment. The terms exhibiting the highest enrichment might help us macroscopically screen possible pathways attributing to the biological reactions of ADSCs in a DCEF, especially those related to the MAPK family. Among all the BP terms, 94 upregulated and 37 downregulated genes related to migration, 80 upregulated and 62 downregulated genes related to proliferation, and 89 upregulated and 76 downregulated genes related to differentiation were screened. mRNAs that were upregulated in the DCEF group, namely, ANXA1, ERRFI1, JAG1, EPHA2, and PRR9, were related to keratinocyte differentiation terms, while H2AFY2 was downregulated. Among the upregulated mRNAs, PRR9 had relatively low expression levels in both groups, indicating that PRR9 may only play a minor role in the process.

For the KEGG pathway analysis, a group of pathways was enriched by upregulated mRNAs after 6 h of EF stimulation. Of these, the TNF signaling pathway, MAPK signaling pathway, and the cytokine-cytokine receptor interaction pathway were prominent. Other related signaling pathways such as the NOD-like receptor, TGF-*β*, estrogen, and the RIG-I-like receptor may also contribute to completing the process of the reaction of ADSCs to the DCEF. As we can see, the term cluster showed that the EF may stimulate the cells, as the signaling pathways triggered are similar to those seen in legionellosis, hepatitis B, influenza A, and salmonella infection (Figure 3(b)).

### 3.4. ceRNA Network Construction

**Figure 4:**
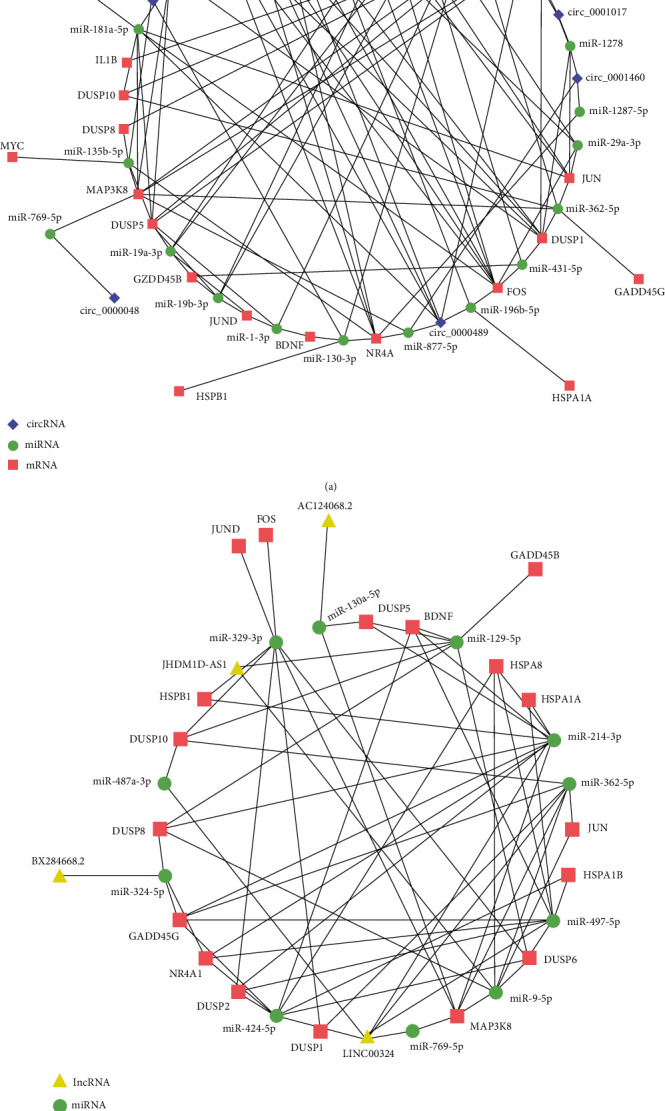
Constructed ceRNA networks based on the selected vital genes related to the MAPK signaling pathway: (a) the circRNA-miRNA-mRNA network and (b) lncRNA-miRNA-mRNA network. Purple diamonds represent circRNAs, yellow triangles represent lncRNAs, green nodes represent miRNAs, and red frames represent mRNAs.

ceRNA networks were constructed based on a selected group of mRNAs. The groups of mRNAs related to the MAPK signaling pathway in upregulated mRNAs were picked to maintain the showed ceRNA network (Figure 4). In this study, 7 circRNAs including hsa_circ_0000489, hsa_circ_0001222, hsa_circ_0001017, hsa_circ_0001460, hsa_circ_0001910, hsa_circ_0000268, and hsa_circ_0000048; 4 lncRNAs including JHDM1D-AS1, AC124068.2, LINC00324, and BX284668.2; 30 miRNAs such as miR-362-5p; and 20 mRNAs were related to the network shown. In the network, miR-362-5p had 12 different regulatory relationships between other circRNA, lncRNA, and mRNA; circ_0000489 could interact with 7 miRNAs, and MAP3K8 could interact with 14 miRNAs. The relationships between these RNAs may provide a novel perspective into the MAPK signaling pathway and the regulation of the proliferation, differentiation, and migration of ADSCs in DCEF.

### 3.5. Validation of circRNA, lncRNA, miRNA, and mRNA Expression

**Figure 5:**
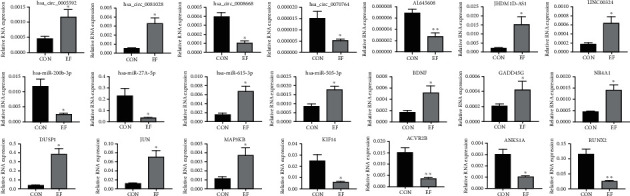
Comparison of the circRNAs, lncRNAs, miRNAs, and mRNAs between the EF and control groups by qRT-PCR. hsa_circ_0005592, hsa_circ_0081028, JHDM1D-AS1, LINC00324, hsa_miR-615-3p, hsa_miR-505-3p, BDNF, GADD45G, NR4A1, DUSP1, JUN, and MAP3KB were upregulated in the EF group. Other RNAs were downregulated in the EF group. The error bars represent the standard deviation of measurements for three replicates in three separate sample runs (*n* = 9; ^∗^*p* < 0.05, ^∗∗^*p* < 0.01).

According to the MAPK signaling pathway, we selected six upregulated mRNAs, namely, BDNF, GADD45G, NR4A1, DUSP1, JUN, and MAP3KB, to validate their expression. Four downregulated mRNAs were chosen, namely, KIF14, ACVR2B, ANKS1A, and RUNX2. Although these mRNAs have few relationships with the MAPK signaling pathway, they were associated with the cell proliferation, migration, and differentiation processes. The four circRNAs, three lncRNAs, and four miRNAs were randomly selected among the mRNAs showing altered expression profiles. All 21 RNAs showed significant differential expression with *p* values of <0.05 (Figure 5).

## 4. Discussion

ADSCs hold significant promise for regenerative medicine due to their ease of harvesting and multilineage differentiation ability. Physical stimulators like EF can promote stem cell differentiation. It has been confirmed that, for ADSCs, 5 min pulses of 448 kHz sine wave current, at a subthermal density of 50 *μ*A/mm^2^, separated by 4 h interpulse lapses, along a total period of 48 h, could enhance cell proliferation rate [15]. Studies have also shown that application of 1 kHz and 2 mV/mm low-frequency ACEF leads to chondrogenesis in ADSCs [16]; application of 1 Hz and 100 mV/mm low-frequency ACEF for 4 h/day for 14 days promoted osteogenic differentiation of ADSCs cultured in osteogenic differentiation medium [17]; application of +4 V and 1 ms and –4 V, 1 ms, and 1 Hz ACEF-treated mouse ADSCs for 72 h showed an induced transcriptional profile more closely related to that of neonatal cardiomyocytes [18]; 35–53 mV/mm EF (current 5 s on, 20 s off) with copper for 1 h can induce differentiation of ADSCs toward the neuronal lineage [19]. From these studies, we could know that different parameters of EF would have different effect on ADSC differentiation, with ACEFs of great importance among them. Studies have also reported the alignment, elongation, and cathode migration of ADSCs in a DCEF [8, 20]. So, whether DCEF can regulate the differentiation of ADSCs into osteoblasts, keratinocytes, and other cell lineages and how a DCEF could regulate changes in cell morphology and migration remains a question for our further studies. Different cells may require different intensities of DCEF to respond. In our preparatory experiments, we tried different intensities of DCEF to treat ADSCs, in the range of 200-600 mV/mm. We recorded that DCEF at 300 mV/mm was strong enough to orient the cells while low rate of cell death (data not shown).

The MAPK signaling pathway map can be roughly divided into the classical MAP kinase pathway, JNK and p38 MAP kinase pathway, and ERK5 pathway. Our 21 upregulated MAPK-related mRNAs seemed to be divided into these three parts. In the classical part, BDNF as a growth factor, DUSPs, FOS, and MYC were mentioned. BDNF participates in the classical MAP kinase pathway by binding to its tyrosine kinase receptor TrkB, leading to cell proliferation and differentiation [21]. Downregulated c-myc could inactivate the p38 MAPK pathway and suppress cell proliferation and migration [22], suggesting that the upregulated c-myc observed in our study may induce these responses. DUSP is a group of dual-specificity phosphatases that are closely related to MAPK and mostly negatively regulate their function. Among the selected MAPK-related genes, DUSP1, DUSP2, and DUSP5 are located in the nucleus, while DUSP6, DUSP8, and DUSP10 are located in the cytoplasm. DUSP1 and DUSP6 dephosphorylate ERK, while DUSP10 is related to JNK/p38 [23]. For the JNK and p38 MAP kinase pathways, IL1B, MAP3K8, HSPB1, DUSP10, FOS, JUN, and JUND were included. IL1B serves as an upstream molecule, while MAP3K8 acts as a MAPKKK. AP-1, JUND, and HSP1B, which also contribute to the MAPK pathway, can be phosphorylated to regulate proliferation, differentiation, and apoptosis. Furthermore, GADD45B and GADD45G could contribute to p38 activation and regulate development and cell apoptosis [24]. HSP70 proteins are also related to JNK and p38 MAPK functions [25]. In the ERK5 pathway, ERK5 enhances the transcriptional activity of NR4A1 through phosphorylation [26]. Then, NR4A1, a transcription factor that regulates cell proliferation and apoptosis, can be induced by a variety of stimuli. The NR4A1 mRNA expression level in the BMSCs of postmenopausal osteoporosis patients was reported to be significantly higher than that in the normal control group, suggesting that NR4A1 may be related to osteogenesis [27]. As a result, the abovementioned MAPK-related genes might make a difference in ADSC homeostasis and proliferation and most interestingly, in differentiation. In the process of epidermal maturation, DUSP6 and other phosphatases promote keratinocyte differentiation by suppressing ERK MAPK and inducing AP-1. However, DUSP10 expression antagonized the process, forming a network to regulate epidermal homeostasis [28]. Also, wound-induced AP-1 plays a pivotal role in fetal skin reepithelialization and keratinocyte differentiation via MAPK [29]. Thus, the EF-induced alterations in the mRNA expression may participate in keratinocyte differentiation, which leads us to investigate this in greater detail. Several dysregulated mRNAs related to keratinocyte differentiation attracted our attention. As the component of the cell envelopes barrier structure of keratinocytes, ANXA1 participates in the process of keratinocyte terminal differentiation [30]. EPHA2, one of the receptor tyrosine kinases, is abundantly expressed in keratinocytes. EPHA2 plays an important role in eliciting the desmoglein 1 expression, enhancing adhesion, and promoting the differentiation of keratinocytes [31]. JAG1, the ligand for multiple Notch receptors, has the ability to induce keratinocyte differentiation and form the stratum corneum, via activating NF-*κ*B and inducing PPARɣ. ERRFI1 is a negative regulator of EGFR signaling. ERRFI1 can regulate keratinocyte differentiation and prevent their overproliferation via the MAPK pathway [32]. All the abovementioned upregulated genes suggested the induction of keratinocyte differentiation. However, H2AFY2 was downregulated in our profile, and this mRNA encodes macroH2A.2, which is a histone variant expressed at low levels in the stem cells but is induced during differentiation and is also related to keratinocyte differentiation [33]. Since this histone may participate in many other processes, H2AFY2 may induce minor effects in differentiation. Due to the number of advantages mentioned before, ADSCs are expected to differentiate into keratinocytes in conditions such as chronic ulcers or other cutaneous disease treatment. Researchers have worked hard to discover methods of inducing ADSCs into keratinocytes, such as coculture with keratinocytes or fibroblasts, or using BMP4 or other biological factors [34–37]. Knowing about such changes of related genes may form the basis for the exploration of EF-induced differentiation of ADSCs to keratinocytes.

To our knowledge, no studies have been published about the transcriptome sequencing and ceRNA network prediction of ADSCs in EF. In this paper, we designed laboratory-made DCEF equipment and treated ADSCs in 0 (control group) or 300 mV/mm EF (DCEF group) for 6 h. ADSCs were identified by flow cytometry, and their multilineage potential was confirmed. We obtained circRNA, lncRNA, miRNA, and mRNA from both groups from 3 donors. By using different ways of screening, standardization, and comparison with databases, the expression profiles and differentially expressed RNAs were analyzed.

Upregulated and downregulated mRNAs were enriched to GO terms and KEGG pathways. Among all the related pathways, the MAPK signaling pathway, which is also an important pathway regulating ADSC proliferation [38], differentiation [39], and migration [40], had the highest gene ratio in the upregulated mRNA enriched pathways. To screen the three terms related to MAPK signaling pathway, all the BP terms enriched by differential expressed mRNAs were calculated (*p* < 0.05). A total of 80 upregulated and 62 downregulated proliferation-related genes were found while there were 94 upregulated and 37 downregulated migration-related genes. Furthermore, 89 upregulated and 76 downregulated genes related to differentiation were found, and the most enriched terms were for fat cell differentiation, cell differentiation, and osteoblast differentiation in the upregulated group and cell differentiation, neuron differentiation, and hematopoietic progenitor cell differentiation in the downregulated group. Four circRNAs, three lncRNAs, and four miRNAs expressed differentially between the EF and control groups were randomly chosen from the pool of differentially expressed RNAs and were validated by RT-PCR. The expression of ten mRNAs related to the MAPK signaling pathway or cell proliferation, migration, and differentiation was also validated.

We constructed ceRNA networks containing circRNAs or lncRNAs of ADSCs in the EF and control groups based on a series of upregulated MAPK signaling pathway mRNAs, which meant the upregulated circRNAs, upregulated lncRNAs, and downregulated miRNAs were used to contribute to the network. Finally, 7 circRNAs, 4 lncRNAs, 30 miRNAs, and 20 mRNAs were included in the network. The prediction of the ceRNA network may shed light on the regulation process of proliferation, migration, and differentiation of ADSCs in DCEF.

## 5. Conclusions

The MAPK signaling pathway is of great importance in the ADSC processes that occur in a DCEF, including keratinocyte differentiation. Several ceRNAs may participate in the regulation of MAPK signaling. This study may give new insight into the proliferation, migration, and differentiation of ADSCs that will be valuable for tissue engineering and regenerative medicine.

## Figures and Tables

**Table 1:** Statistical analysis of all differentially expressed ncRNAs and mRNAs.

Differential expressed RNAs	Total no.	No. of upregulated	No. of downregulated	The most upregulated (log_2_ FC)	The most downregulated (log_2_ FC)
circRNA	66	38	28	hsa_circ_0008650 (5.65)	hsa_circ_0001582 (-5.19)
lncRNA	164	24	140	AK298056 (5.53)	BX538221_2 (-4.38)
miRNA	26	5	21	hsa-miR-novel-chr7_23770 (7.72)	hsa-miR-novel-chr17_8249 (-4.83)
mRNA	1310	274	1036	FOS (7.10)	DNMT3B (-10.75)

## Data Availability

Sequencing data have been submitted to the NCBI Gene Expression Omnibus, accession number GSE149888.

## Supplementary Materials

Supplementary Materials Library preparation and RNA sequencing RNA libraries were constructed from total RNA using NEBNext® Ultra™ II Directional RNA Library Prep Kit (New England Biolabs, Inc.). The quality of the library was controlled and quantified using a BioAnalyzer 2100 system (Agilent Technologies, Inc., USA). Library sequencing was performed on an llumina Hiseq 4000 sequencer; then, paired-end reads were harvested. High-quality trimmed reads were acquired after quality control by Q30 and 3′-adaptor-trimming together with removal of low-quality reads. Identification of RNAs and differential expression analysis lncRNAs and mRNAs: high-quality reads were aligned to the human reference genome (UCSC HG19). The fragments per kilobase of exon per million fragments mapped (FPKM) values of the lncRNA and mRNA expression profiles were obtained under the guidance of the GTF gene annotation file. The fold change and **p** values of the EF and control groups were calculated and compared to screen for differentially expressed lncRNAs and mRNAs. Fold change ≥ 2.0 (i.e., log 2 (FC) ≥ 1.0, **p** value < 0.05) and FPKM value of ≥0.1 in at least one sample were used as the threshold values when screening of the differential expression. CircRNAs: high-quality reads were aligned to the reference genome/transcriptome. CircRNAs were detected and identified. CircBase and Circ2Traits were used to annotate the identified circRNAs. Then, edgeR was used for data standardization and differential expression of circRNAs. Differential expression of circRNA was determined as a fold change of ≥2.0 and a **p** value < 0.05. MiRNAs: total RNA was prepared to establish the miRNA sequencing library, including 3′-adaptor ligation, 5′-adaptor ligation, cDNA synthesis, and PCR amplification. The libraries were denatured as single-stranded DNA molecules, captured on an Illumina flow cell, amplified as clusters in situ, and then sequenced. After sequencing, image analysis, base recognition, and quality control using Q30, raw data were generated. The trimmed reads were aligned to the merged human pre-miRNA databases (miRBase and the predicted pre-miRNAs) with at the most one mismatch. After defining the raw expression levels of the miRNA, the raw counts were normalized by edgeR and filtered to find differentially expressed miRNAs with a fold change of ≥1.5 and a **p** value of <0.05. Table S1: the primer sequences of 21 validated RNAs.
