# Supplementary material for: Changing Expression Profiles and Inclination to Competing Endogenous RNA Networks on MAPK Signaling Pathways of Human Adipose-Derived Stem Cells in a Direct Current Electric Field
Source: Biomed Res Int. 2020 Nov 6;2020:7134719. doi: 10.1155/2020/7134719 (PMC7666630; doi:10.1155/2020/7134719)
Supplement: Supplementary Materials — Library preparation and RNA sequencing RNA libraries were constructed from total RNA using NEBNext® Ultra™ II Directional RNA Library Prep Kit (New England Biolabs, Inc.). The quality of the library was controlled and quantified using a BioAnalyzer 2100 system (Agilent Technologies, Inc., USA). Library sequencing was performed on an llumina Hiseq 4000 sequencer; then, paired-end reads were harvested. High-quality trimmed reads were acquired after quality control by Q30 and 3′-adaptor-trimming together with removal of low-quality reads. Identification of RNAs and differential expression analysis lncRNAs and mRNAs: high-quality reads were aligned to the human reference genome (UCSC HG19). The fragments per kilobase of exon per million fragments mapped (FPKM) values of the lncRNA and mRNA expression profiles were obtained under the guidance of the GTF gene annotation file. The fold change and p values of the EF and control groups were calculated and compared to screen for differentially expressed lncRNAs and mRNAs. Fold change ≥ 2.0 (i.e., log 2 (FC) ≥ 1.0, p value < 0.05) and FPKM value of ≥0.1 in at least one sample were used as the threshold values when screening of the differential expression. CircRNAs: high-quality reads were aligned to the reference genome/transcriptome. CircRNAs were detected and identified. CircBase and Circ2Traits were used to annotate the identified circRNAs. Then, edgeR was used for data standardization and differential expression of circRNAs. Differential expression of circRNA was determined as a fold change of ≥2.0 and a p value < 0.05. MiRNAs: total RNA was prepared to establish the miRNA sequencing library, including 3′-adaptor ligation, 5′-adaptor ligation, cDNA synthesis, and PCR amplification. The libraries were denatured as single-stranded DNA molecules, captured on an Illumina flow cell, amplified as clusters in situ, and then sequenced. After sequencing, image analysis, base recognition, and quality control using Q30, raw data were [file 7134719.f1.docx]

Supplementary Methods:

Library preparation and RNA sequencing

RNA libraries were constructed from total RNA using NEBNext® Ultra™ II Directional RNA Library Prep Kit (New England Biolabs, Inc.). The quality of the library was controlled and quantified using a BioAnalyzer 2100 system (Agilent Technologies, Inc., USA). Library sequencing was performed on an llumina Hiseq 4000 sequencer, then paired-end reads were harvested. High-quality trimmed reads were acquired after quality control by Q30, and 3ʹ-adaptor-trimming together with removal of low-quality reads.

Identification of RNAs and differential expression analysis

LncRNAs and mRNAs: High-quality reads were aligned to the human reference genome (UCSC HG19). The fragments per kilobase of exon per million fragments mapped (FPKM) values of the lncRNA and mRNA expression profiles were obtained under the guidance of the GTF gene annotation file. The fold change and p-values of the EF and control groups were calculated and compared to screen for differentially expressed lncRNAs and mRNAs. Fold change ≥ 2.0 (i.e., log 2(FC) ≥ 1.0, p-value < 0.05) and FPKM value of ≥ 0.1 in at least one sample were used as the threshold values when screening of differential expression.

CircRNAs: High-quality reads were aligned to the reference genome/transcriptome. CircRNAs were detected and identified. CircBase and Circ2Traits were used to annotate the identified circRNAs. Then, edgeR was used for data standardization and differential expression of circRNAs. Differential expression of circRNA was determined as a fold change of ≥ 2.0 and a p-value < 0.05.

MiRNAs: Total RNA was prepared to establish the miRNA sequencing library, including 3ʹ-adaptor ligation, 5ʹ-adaptor ligation, cDNA synthesis and PCR amplification. The libraries were denatured as single-stranded DNA molecules, captured on an Illumina flow cell, amplified as clusters in situ and then sequenced. After sequencing, image analysis, base recognition, and quality control using Q30, raw data were generated. The trimmed reads were aligned to the merged human pre-miRNA databases (miRBase and the predicted pre-miRNAs) with at the most one mismatch. After defining the raw expression levels of the miRNA, the raw counts were normalized by edgeR, and filtered to find differentially expressed miRNAs with a fold change of ≥1.5 and a p-value of <0.05.

Table S1: the primer sequences of 21 validated RNAs.

| circ_0005592 | Forward | GGAGCCCAAAATTGCAGAGC |
| --- | --- | --- |
|  | Reverse | GGCCAGACATGTAGCGATCA |
| circ_0081028 | Forward | GATGAGAAGGCCTGTGTGCT |
|  | Reverse | ACCACATGGGAACATGGCTT |
| circ_0008668 | Forward | CATCGCCCAGGATGACTCAA |
|  | Reverse | GATGGGCTCCTGATGTCTCG |
| circ_0070764 | Forward | GCGGTACATTTCTACAACAGCC |
|  | Reverse | TCCTCCTTGGCACTTTTCTCC |
| AL645608 | Forward | CCACACGCTGTTTAAGACCC |
|  | Reverse | TCAGATGAACAGGCAGAGGG |
| JHDM1D-AS1 | Forward | CCTTGAAATGGGGTTGGTGG |
|  | Reverse | TACCCCATCTTCCCCACTTG |
| LINC00324 | Forward | AGCATCTCAATCCCCAGACC |
|  | Reverse | TCAGGAAGCAGAAAGGGAGG |
| hsa-miR-200b-3p | Reverse Transcription | GTCGTATCCAGTGCGTGTCGTGGAGTCGGCAATTGCACTGGATACGACTCATCA |
|  | Forward | GGGTAATACTGCCTGGTAA |
|  | Reverse | CAGTGCGTGTCGTGGAGT |
| hsa-miR-27a-5p | Reverse Transcription | GTCGTATCCAGTGCGTGTCGTGGAGTCGGCAATTGCACTGGATACGACTGCTCA |
|  | Forward | GGGAGGGCTTAGCTGCTTG |
|  | Reverse | CAGTGCGTGTCGTGGAGT |
| hsa-miR-615-3p | Reverse Transcription | GTCGTATCCAGTGCGTGTCGTGGAGTCGGCAATTGCACTGGATACGACAAGAGG |
|  | Forward | GGGTCCGAGCCTGGGTCTC |
|  | Reverse | CAGTGCGTGTCGTGGAGT |
| hsa-miR-505-3p | Reverse Transcription | GTCGTATCCAGTGCGTGTCGTGGAGTCGGCAATTGCACTGGATACGACAGGAAA |
|  | Forward | GGGCGTCAACACTTGCTGG |
|  | Reverse | CAGTGCGTGTCGTGGAGT |
| U6 | Reverse Transcription | AACGCTTCACGAATTTGCGT |
|  | Forward | CTCGCTTCGGCAGCACA |
|  | Reverse | AACGCTTCACGAATTTGCGT |
| BDNF | Forward | TGGAGGCTATGTGGAGTTGG |
|  | Reverse | CACCTCCACCTAGACCTTGG |
| GADD45G | Forward | CACAGTTCCGGAAAGCACAG |
|  | Reverse | CAGAAGGTCACATTGTCGGG |
| NR4A1 | Forward | GGGCATGGTGAAGGAAGTTG |
|  | Reverse | AGGGAAGTGAGGAGATTGGC |
| DUSP1 | Forward | ACTTCACCCGAGTTCCTCTG |
|  | Reverse | AGTCCTTTCTCTTCTGCCCC |
| JUN | Forward | CAACATGCTCAGGGAACAGG |
|  | Reverse | GTTAGCATGAGTTGGCACCC |
| MAP3K8 | Forward | GGCCATTCAACCAAAGCAGA |
|  | Reverse | GATAGGCTGAGCGAGGGTAG |
| KIF14 | Forward | CTGCTCTACGGCTCACACTA |
|  | Reverse | GCTTGTTCCGAAAGTGCAGA |
| ACVR2B | Forward | GAAGGCCCAGCTCATGAATG |
|  | Reverse | TGTGCTGAAGATCTCCCGTT |
| ANKS1A | Forward | GCAACTCAAAGGTTTCCGGT |
|  | Reverse | CACTGACAGGTAAGAGGCCA |
| RUNX2 | Forward | AGTCAGGGTCCCTTCATTGG |
|  | Reverse | AAGGACCAGAGAACAAGGGG |
| GAPDH | Forward | GGCCTCCAAGGAGTAAGACC |
|  | Reverse | AGGGGAGATTCAGTGTGGTG |
